# Mechanisms and Research Progress of LncRNA-Mediated Regulation of Viral Infections

**DOI:** 10.3390/v18101141

**Published:** 2026-10-14

**Authors:** Menghua Peng, Fenfang Luo, Ning Li

**Affiliations:** 1 College of Animal Science and Technology, Jiangxi Agricultural University, Nanchang 330045, China; jxncpengmenghua@163.com; 2 Nanchang Police Dog Base of Ministry of Public Security, Nanchang 330100, China; 3 Pet Medical Teaching and Research Office, Department of Animal Science, Jiangxi Biotech Vocational College, Nanchang 330200, China; 4 Key Laboratory of Animal Pathogen Infection and Immunology of Fujian Province, College of Animal Science, Fujian Agriculture and Forestry University, Fuzhou 350002, China

**Keywords:** long non-coding RNA, antiviral immunity, programmed cell death

## Abstract

Long non-coding RNAs (lncRNAs), a class of RNA molecules whose transcripts exceed 200 nucleotides in length and typically lack protein-coding capacity, were once widely regarded as “transcriptional noise.” However, recent studies have shown that lncRNAs can encode small peptides and play critical roles in biological processes, including gene expression regulation, cell differentiation, and immune response. Viral infections, as a common disease, can reshape the transcriptome of host cells, in which the differential expression of lncRNAs is a core link in host–virus interactions. This review aims to deeply analyze the multidimensional regulatory mechanisms of lncRNAs in the host antiviral innate immune response. It elaborates in detail on how lncRNAs construct the host antiviral defense by regulating pattern recognition receptor (PRR) signaling pathways, the production and signal transduction of interferons (IFNs), as well as the expression of interferon-stimulated genes (ISGs). Additionally, we conducted an in-depth analysis of the complex functions of lncRNAs in regulating programmed cell death (PCD), including key processes such as pyroptosis, autophagy, and apoptosis, and integrated specific research cases in infections caused by multiple significant viruses, including influenza A virus (IAV), human immunodeficiency virus (HIV), hepatitis B virus (HBV), hepatitis C virus (HCV), and severe acute respiratory syndrome coronavirus 2 (SARS-CoV-2). We aim to provide novel strategies for the diagnosis, treatment, and prevention of viral diseases.

## 1. Introduction

Viral infectious diseases have long posed a threat to global public health and socioeconomic stability. From the Spanish flu, SARS, and MERS to influenza A (H1N1), Ebola, and the COVID-19 pandemic caused by the novel coronavirus, viruses continue to challenge human health and security through their high mutability, rapid transmission, and immune evasion capabilities [1,2,3]. Although vaccines and antiviral drugs have controlled the epidemics to a certain extent, drug resistance and immune evasion resulting from viral mutations have made it increasingly difficult to develop broad-spectrum and highly effective prevention and treatment strategies. Traditional antiviral drugs predominantly target viral enzymes (such as reverse transcriptase, protease, and polymerase), and are limited by a single target, susceptibility to drug resistance, and host toxicity. Therefore, the in-depth dissection of the molecular network of host–virus interactions and the discovery of host-derived defense pathways that viruses cannot easily hijack have become a critical breakthrough point for the development of next-generation antiviral drugs. Through long-term evolution, host cells have developed a precise and robust innate immune system, which serves as the first key line of defense against viral invasion. This system recognizes pathogen-associated molecular patterns through pattern recognition receptors, activates downstream signaling cascades, induces the production of interferons and pro-inflammatory cytokines, and thereby upregulates the expression of hundreds of interferon-stimulated genes, establishing an antiviral state [4,5,6]. In this complex and orderly process, protein-coding genes are certainly important, but non-coding RNAs, which constitute the vast majority of transcripts in the human genome, especially long non-coding RNAs, as key members of regulatory networks, are increasingly showing their critical roles. lncRNAs, with their complex expression patterns and diverse regulatory mechanisms, are deeply involved in host antiviral immune responses and viral pathogenesis, providing new perspectives and potential targets for antiviral research.

LncRNAs are primarily transcribed by RNA polymerase II [7], possess mRNA-like structures including a 5′ cap and 3′ poly(A) tail [8] and exhibit tissue specificity and extensive alternative splicing features [9]. They originate from various genomic mechanisms, including the conversion of protein-coding sequences [10], chromosomal rearrangements [11,12], retrotransposition [13], local tandem duplication [14], and transposable element insertion [15]. Functionally, lncRNAs function as signals, decoys, and other pathways to regulate gene transcription and protein modification [16,17]. Previous scholars believed that lncRNAs lack an open reading frame and therefore do not possess protein-coding capacity. However, recent studies have found that short open reading frames (sORFs) in lncRNAs can encode functional peptides. An lncRNA can contain one or more sORFs, which can be translated into small peptides of fewer than 100 amino acids, although many transcripts encoding functional peptides may have been misannotated as “non-protein-coding” [18,19]. These lncRNA-encoded peptides have also been demonstrated to play critical roles in various physiological and pathological processes, especially during tumorigenesis and pathogenic microbial infection [20,21]. These micropeptides exert their functions by regulating biological processes such as inflammatory immunity, mitochondrial metabolism, and Ca2+ transport, and hold great potential in clinical diagnosis, prognosis evaluation, and the discovery of new drug targets [22].

During viral infection, the expression profiles of host lncRNAs undergo significant reprogramming, forming a complex virus–host interaction network. Some induced lncRNAs exert antiviral effects by activating innate immune pathways, directly suppressing viral replication, or inducing host cell apoptosis, thereby establishing an intrinsic defense barrier. Conversely, other lncRNAs are hijacked by viruses to promote viral replication or suppress host immune responses, favoring persistent viral infection and pathogenesis. Therefore, a systematic understanding of the functions of lncRNAs in antiviral immunity not only deepens our insight into the mechanisms of host–pathogen coexistence but also provides new perspectives and potential therapeutic targets for addressing emerging viral threats.

## 2. Multidimensional Regulation of Viruses by Long Non-Coding RNA

The host innate immune system is a complex regulatory network characterized by multi-level and finely tuned regulation. Its core function is to recognize invading pathogens and initiate effective defensive responses. In this process, lncRNAs can serve as key regulatory factors (Table 1), acting at different stages through multiple mechanisms. They can positively or negatively regulate relevant signaling pathways, balancing the intensity and duration of antiviral responses, thereby avoiding severe tissue damage or persistent viral infection.

### 2.1. Regulation of PRR Signaling Pathways by LncRNA

When a virus enters the host, pathogen-associated molecular patterns (PAMPs) are recognized by pattern recognition receptors (PRRs), including Toll-like receptors (TLRs) and RIG-I-like receptors (RLRs). These receptors transmit signals through adaptor proteins, thereby initiating downstream inflammatory and antiviral responses. Accumulating evidence indicates that lncRNAs can actively regulate these PRR signaling pathways through multiple molecular mechanisms. For example, lnc-NEAT1 enhances TLR3 stimulation-induced CXCL8 expression in HeLa and A549 cells by binding to the splicing factor SFPQ and promoting its dissociation from the CXCL8 promoter region [23]. Lnc-ITPRIP-1 positively regulates the RLR signaling pathway by targeting MDA5, thereby inhibiting hepatitis C virus (HCV) replication [24]; and lnc-ZC3H7A enhances RIG-I-mediated antiviral defense via TRIM25-dependent K63 ubiquitination of RIG-I [25]. These examples illustrate the functional diversity of lncRNAs at distinct nodes of PRR pathways. Systematic transcriptomic profiling has further expanded our understanding of lncRNA involvement in PRR signaling. In a study of duck hepatitis A virus type 1 (DHAV-1) infection, Sui et al. [26] identified thousands of differentially expressed lncRNAs through RNA sequencing, among which cis- and trans-target analyses implicated multiple lncRNAs in the TLR, NOD-like receptor (NLR), RLR, and cytosolic DNA-sensing pathways; notably, six lncRNAs—including lnc-XR_001188764.3 and lnc-TCONS_00161164—were predicted to target the TLR pathway, and one of them, lnc-TCONS_00113417, showed significantly increased expression upon DHAV-1 infection, correlating with upregulated expression of its target genes IL-6 and IL-10, both of which are key downstream effectors of TLR signaling and critical mediators of host antiviral immunity [26,55].

Among the most extensively characterized lncRNAs in antiviral immunity is NEAT1, a confirmed positive regulator of the IL-8 signaling pathway. NEAT1 expression is induced by influenza virus and HIV infections, leading to the formation of nuclear paraspeckles [27,28]. Mechanistically, NEAT1 mediates the relocalization of the IL-8 negative regulator SFPQ from the IL-8 promoter to paraspeckles, thereby derepressing IL-8 transcription; by knocking out or inactivating ALKBH5, the researchers found that hypoxia-induced tumor-associated macrophage recruitment and immunosuppression, as well as CXCL8/IL8 expression and secretion, were significantly inhibited [29]. In addition, NEAT1 serves as a scaffold that maintains paraspeckle integrity and sequesters viral mRNA in the nucleus during HIV infection, thus inhibiting viral replication [30]. Researchers have for the first time screened and identified the lncRNA NEAT1 that binds to the NLRP3 inflammasome through CLIP-seq, and confirmed that it directly participates in the assembly of multiple inflammasomes, including NLRP3, NLRC4, and AIM2, filling the research gap regarding the direct regulation of inflammasome assembly by lncRNAs [31].

Another functionally important lncRNA is lncRNA-DC, a dendritic cell (DC)-specific transcript that is activated upon hepatitis B virus (HBV) infection [56]. Lnc-DC modulates DC growth, apoptosis, and immune responses through the TLR9/STAT3 signaling axis; specifically, it reduces secretion of TNF-α, IL-6, IL-12, and IFN-γ while increasing IL-1β production, and promotes DC maturation via STAT3 phosphorylation [32]. Beyond their mechanistic roles, these lncRNAs have also shown clinical relevance. ROC curve analyses have revealed that serum levels of NEAT1 and lncRNA-DC may serve as predictive biomarkers for Behçet’s disease, with high NEAT1 levels significantly correlating with negative laboratory findings and high lncRNA-DC levels with positive family history [33].

### 2.2. LncRNA Mediates IRF3 Phosphorylation to Regulate IFN Expression

Data indicate that host pattern recognition receptors recognize nucleic acid components of invading viruses and are subsequently activated by signaling adaptors (such as the adaptor containing the TLR3-TIR domain) to recruit and activate TANK-binding kinase 1 (TBK1), which then phosphorylates interferon regulatory factor IRF3. Upon phosphorylation, IRF3 forms homodimers and subsequently translocates into the nucleus, where it cooperates with other transcription factors (such as IRF7) to initiate the induction of IFN-stimulated genes (ISGs) by IFN (mainly type I IFN), thereby protecting the host from viral infection [57]. Several lncRNAs directly modulate IRF3 activity. Xu et al. [34] used FLAG tagging, immunoprecipitation, and plasmid construction methods to find that after vesicular stomatitis virus (VSV) infects the host, IFN-I induces the expression of lncRNA-ISIR and IRF3, accelerates the dissociation of IRF3 from Fli-1, further enhances the efficiency of the innate immune response, and inhibits viral replication. Conversely, the nuclear-enriched lncRNA Malat1 negatively regulates type I IFN production. After VSV infection, lncRNA Malat1 directly binds to the reactive DNA-binding protein (TDP43) in the nucleus and prevents TDP43 activation by blocking the activated caspase-3-mediated cleavage of TDP43 to TDP35. The cleaved TDP35 enhances IRF3 protein levels by binding to and degrading Rbck1 pre-mRNA, thereby preventing IRF3 proteasomal degradation during viral infection, which selectively promotes antiviral type I IFN production. Lack of Malat1 enhances antiviral innate responses in vivo, accompanied by increased type I IFN production and reduced viral replication [35]. LncRNAs also target upstream kinases in the IFN pathway. lncRNA-GM binds to glutathione S-transferase M1 (GSTM1), blocking its interaction with TBK1 and reducing GSTM1-mediated S-glutathionylation of TBK1. Decreased S-glutathionylation can enhance TBK1 activity and the production of downstream antiviral mediators, and inhibit viral replication [36].

ISGs can be induced by interferon signaling pathways following viral infection, which is crucial for efficient antiviral responses and the restoration of cellular homeostasis. However, the molecular mechanisms regulating ISG expression are highly complex, and studies have shown that lncRNAs are key regulators of ISG expression. lncRNAs modulate ISG expression by altering the function of viral sensors and the production of IFN. For example, lnc-ATV [37] and lncRNA-LSm3b [38] interact with the cytosolic dsRNA sensor RIG-I and restrict its function, whereas lnc-ZC3H7A promotes RIG-I activity by facilitating its interaction with TRIM25 [25]. lnc-ISG20 functions as a competitive endogenous RNA (ceRNA) that sponges miR-326, thereby relieving miR-326-mediated suppression of ISG20 translation and inhibiting IAV replication [39]. Additionally, lncRNA-155, a derivative of mir-155hg, directly regulates host innate immunity during viral infection by modulating IFN responses mediated by TLR3 and protein tyrosine phosphatase 1B (PTP1B) [40]. Some viruses can induce the expression of lncRNA-BISPR through the JAK-STAT pathway, such as influenza virus, vesicular stomatitis virus, or hepatitis C virus [41,42]. lncRNA-BISPR is located adjacent to the ISG BST2, whose encoded protein tethers viruses to the cell surface and inhibits viral release. Mechanistically, lncRNA-BISPR attenuates the repressive effect of the polycomb repressive complex 2 (PRC2) on the BST2 promoter, thereby upregulating BST2 expression [58]. Beyond its mechanistic role, researchers used qPCR to detect the expression levels of lncRNA GAS5, lncRNA HEIH, lncRNA BISPR, and mRNA BST2 in the serum of HCV-infected patients and healthy controls, and found that long non-coding RNAs GAS5, HEIH, BISPR, and their mRNA BST2 are closely associated with HCV infection and its antiviral response, demonstrating potential as novel non-invasive biomarkers for the diagnosis and prognosis of HCV GT4 infection [43].

### 2.3. LncRNA Regulates Programmed Cell Death

Programmed cell death (PCD) is a normal physiological response that occurs in cells under the regulation of their own genes and multiple factors. According to the mechanisms and biochemical and morphological characteristics, PCD includes various forms such as apoptosis, ferroptosis, pyroptosis, and autophagy. Accumulating evidence has implicated lncRNAs in the regulation of PCD, primarily in the context of tumorigenesis and inflammatory diseases. For instance, lncRNA-EIF3J-DT [44], lncRNA NBR2 [45], and lncRNA DDIT4-AS1 [46] modulate tumor progression through autophagy regulation, whereas lncRNA-TUG1 [47], lncRNA XIST [48], and lncRNA Gm15834 [49] influence myocardial injury via pyroptosis or autophagic pathways. Despite extensive characterization of lncRNA–PCD crosstalk in sterile inflammation and cancer, their roles in the context of viral infection remain incompletely understood. Nevertheless, emerging evidence indicates that host lncRNAs actively modulate viral replication by regulating autophagy- and pyroptosis-related pathways. These regulatory axes can be broadly categorized into two modes: direct modulation of PCD gene expression, and indirect regulation through upstream signaling axes. In the first mode, lncRNAs directly induce the expression of PCD-related genes. Following coxsackievirus B3 (CVB3) infection, lncRNA ROR binds to PTBP1, destabilizes FOXP1 mRNA, and promotes the expression of IL-1β and IL-18 as well as the release of pyroptotic effectors—including NLRP3, ASC, and cleaved caspase-1—thereby enhancing pyroptosis [50]. In the second mode, lncRNAs act through PCD-related signaling axes. For example, Cao et al. [51] demonstrated in a hepatitis B virus (HBV) cell model that lncRNA WAC-AS1 regulates viral replication by enhancing autophagy through the miR-192-5p/Agt7 signaling axis. Similarly, lncRNA KCNQ1OT1 promotes pyroptosis in HCV-infected β cells via the miRNA-223-3p/NLRP3 axis [52]; notably, KCNQ1OT1 also mediates autophagy induction through the miR-26a-5p/Atg12 axis in ischemia–reperfusion injury [53], highlighting its dual role in distinct PCD pathways.

Beyond its regulatory functions in PCD signaling, KCNQ1OT1 has been shown to modulate chromatin structure and epigenetic states. Using RNA sequencing and proteomics approaches, researchers have revealed that KCNQ1OT1 pairs with double-stranded genomic DNA through sequence-specific base pairing via its repeat-rich regions, and binds to the heterochromatin protein HP1α. It is noteworthy that inhibiting KCNQ1OT1 expression or deleting its specific regions reduces DNA methylation at targeted mobile elements, indicating that KCNQ1OT1 plays a critical role in maintaining genomic stability and regulating cellular senescence [54]. These findings suggest that KCNQ1OT1 may participate in the regulation of PCD in coordination with broader epigenetic regulatory mechanisms, but the intersection of these two functions during viral infection requires further investigation.

## 3. The Role of lncRNA in Different Viral Infections

**Figure 1:**
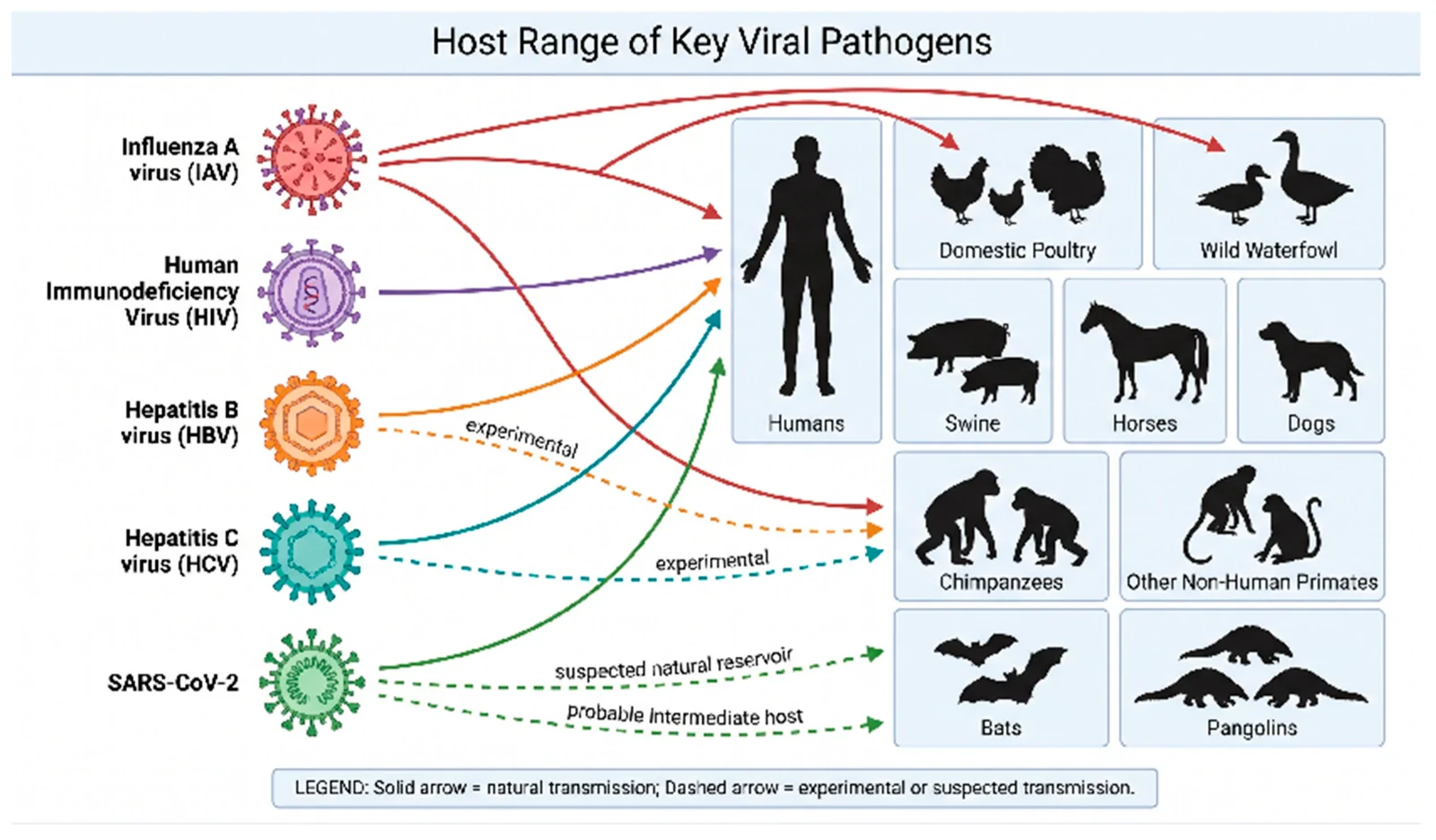
The host range of viral infections.

Influenza A virus (IAV), human immunodeficiency virus (HIV), hepatitis viruses, and severe acute respiratory syndrome coronavirus 2 (SARS-CoV-2) pose a major threat to global public health security; the current literature indicates that the host range of these viral infections is as shown in Figure 1 [59,60,61,62,63,64,65,66]. Moreover, increasing evidence suggests that host and viral long non-coding RNAs (lncRNAs) play a crucial role in disease processes such as viral invasion and replication (Table 2). Given the multiple roles of lncRNAs in these viral systems, we analyze their functions to provide guidance for the development of future antiviral strategies.

### 3.1. Influenza A Virus (IAV)

Influenza A virus (IAV) is an RNA virus with high mutability, posing ongoing challenges to the development of vaccines and antiviral drugs [87]. Numerous studies have revealed that host lncRNAs play a dual role in IAV infection. Some lncRNAs exert protective functions by restricting viral replication, while others are exploited by the virus to promote viral spread [88,89]. In terms of protection, the host initiates antiviral defense mechanisms by upregulating or downregulating specific lncRNAs. For example, lncRNA-IVRPIE is significantly upregulated after IAV infection, and its overexpression can inhibit viral replication in A549 cells. Mechanistically, the researchers used RNA pull-down and qPCR to verify that IVRPIE positively promotes the transcription of type I interferon (IFNβ) and the expression of multiple key ISGs (such as IRF1, IFIT1, and MX1) by regulating histone modifications in the promoter regions of target genes, thereby enhancing host antiviral capacity [67]. Similarly, several other lncRNAs, such as LINC02574 and lncRNA SAAL, have been identified as host factors that restrict IAV replication; It is worth noting that LINC02574 is the first to be reported for its role in IAV infection, and it was found to be induced through the RIG-I/IFN/IFNAR pathway and to positively regulate innate immunity [68,69,70]. In contrast to previous reports that LINC02574 promotes HIV replication, this reveals that the same lncRNA exhibits opposite functions in different viral infections [90].

However, there is also evidence that host lncRNAs do not always exert antiviral effects; certain lncRNAs are instead exploited by viruses to promote infection. This phenomenon is particularly noteworthy in the field of lncRNA-encoded peptides: some peptides encoded by host lncRNAs have been demonstrated to enhance viral replication rather than inhibit infection. Taking PCBP1-AS1 as an example, the small protein PESP encoded by it is induced during IAV infection and enhances autophagy by upregulating ATG7, thereby promoting viral replication [71]. And researchers have discovered for the first time that GAS5-P, a novel 50-amino-acid micropeptide encoded by the classical tumor-suppressive lncRNA GAS5, is significantly upregulated during IAV infection and exhibits proviral function, thereby expanding the functional repertoire of GAS5. Mechanistically, GAS5-P50 interacts with NOTUM, a negative regulator of the Wnt signaling pathway, enhancing activation of the Wnt/β-catenin pathway and thus promoting viral replication [72]. These findings reveal the complex interplay between host lncRNAs and IAV, and highlight the potential of lncRNA-targeting approaches as novel anti-influenza therapeutic strategies.

### 3.2. Human Immunodeficiency Virus (HIV)

Human immunodeficiency virus (HIV), member of the Retroviridae family, destroys the host’s immune function and ultimately leads to acquired immunodeficiency syndrome (AIDS) [91]. Although current anti-HIV drugs can effectively suppress the virus, they cannot completely eliminate the latent virus in the host [92]. Existing data indicate that host lncRNAs can regulate HIV at multiple stages. For example, lncRNA-NEAT1 promotes HIV transcription by interacting with key components of the HIV transcriptional complex, whereas lncRNA NKILA negatively regulates HIV transcription by inhibiting the NF-κB signaling pathway [73,74]. The inability to eradicate the virus is a major obstacle to the treatment of this disease, as it enables HIV to evade immune therapy and host immune surveillance. Researchers employed multiple experimental techniques both in vivo and in vitro (such as ELISA and Western blot) to verify that host lncRNA-TUG1 undergoes altered transcriptional levels after HIV infection and is involved in regulating T-cell activation and the stability of the latent viral reservoir [75]. TUG1 can also mediate the astrocyte senescence process induced by the HIV-1 Tat protein, which is closely associated with HIV-associated neurocognitive disorders [76,77,78].

HIV infection can significantly alter the lncRNA expression network in T cells. These differentially expressed lncRNAs may influence T cell survival and function through programmed cell death and signaling pathways, and are associated with the pathogenesis of HIV-related malignancies [93]. Beyond their roles in infected individuals, specific lncRNAs may confer intrinsic resistance to HIV acquisition. For example, researchers analyzed HESN individuals and control individuals through RNA sequencing, identified potential lncRNAs, and performed functional experimental validation in cell models, finding that LINC02453 is highly expressed in HIV-exposed but seronegative individuals and inhibits HIV-1 replication by binding to the nuclear pore component SEC13, thereby blocking late reverse transcription, nuclear import, and DNA integration—revealing a novel host defense mechanism against HIV infection [79].

### 3.3. Hepatitis B/C Virus (HBV/HCV)

Hepatitis C virus (HCV)—a positive-sense RNA virus of the genus Hepacivirus within the family Flaviviridae—establishes chronic infection that can progress to liver fibrosis, cirrhosis, and hepatocellular carcinoma (HCC) [94]. Similar to HIV, host lncRNAs exert complex regulatory functions throughout the HCV life cycle and are intimately linked to the pathogenesis of HCV-associated liver diseases. A number of lncRNAs are differentially expressed upon HCV infection, functioning either to enhance or to restrain host antiviral immunity and thereby modulating HCV replication. For example, lncRNA-NEAT1—a major structural component of nuclear paraspeckles—is upregulated in response to HCV infection and promotes HCV-induced hepatocarcinogenesis through regulation of the miR-9-5p/BGH3 axis [80]. In addition, certain lncRNAs directly participate in the regulation of HBV replication: researchers compared serum samples from women with HBV infection during pregnancy and non-pregnancy, and laboratory-related experiments showed that lncRNA WAC-AS1 enhances HBV replication by promoting miR-192-5p/ATG7-mediated autophagy [51]. In HBV-related HCC, multiple lncRNAs—including HULC, HOTAIR, and MEG3—are aberrantly expressed and contribute to virus-induced hepatocarcinogenesis through modulation of host gene expression, activation of oncogenes, or suppression of tumor suppressors [81]. Collectively, these findings underscore the pivotal roles of lncRNAs in virus-associated liver disease pathogenesis and highlight their potential as therapeutic targets for HCV and HBV infections.

### 3.4. Severe Acute Respiratory Syndrome Coronavirus 2 (SARS-CoV-2)

Since its emergence in late 2019, severe acute respiratory syndrome coronavirus 2 (SARS-CoV-2) has caused a global pandemic. The resulting coronavirus disease 2019 (COVID-19) is primarily characterized by respiratory symptoms and can even lead to death. Increasing evidence indicates that the expression profiles of host lncRNAs are closely associated with disease severity and clinical prognosis. Multiple independent studies have consistently found that the expression of lncRNA-NEAT1 and lncRNA-MALAT1 is significantly elevated in the blood and bronchoalveolar lavage fluid of COVID-19 patients, and that they participate in the cytokine storm induced by COVID-19 through regulating inflammatory responses [82,83]. Furthermore, researchers used techniques such as qPCR to evaluate the circulating levels of long non-coding RNAs (lncRNAs) ANRIL, THRIL, NEAT1, and MALAT1 in the blood of SARS-CoV-2-infected patients and healthy individuals, and found that the expression levels of ANRIL and THRIL are associated with the severity of COVID-19 and can serve as circulating biomarkers for disease progression [84]. At the molecular level, bioinformatics analysis indicated that the SARS-CoV-2 genome (especially its 5′end region) may interact with multiple host lncRNAs, including HOTAIRM1 and PVT1, thereby potentially influencing viral replication or host immune responses [85]. For example, people report a computational study demonstrating that lncRNA H19 binds to the 5′UTR of the viral genome and, more specifically, to the transcript of the viral gene Spike, which has a pivotal role in viral infection [86]. Although these interactions need to be experimentally validated, they provide new insights into the molecular basis of SARS-CoV-2 pathogenesis. Overall, these findings not only deepen our understanding of SARS-CoV-2 pathogenicity and disease progression, but also open new avenues for therapeutic intervention, including drug strategies targeting lncRNAs.

## 4. Summary and Outlook

**Figure 2:**
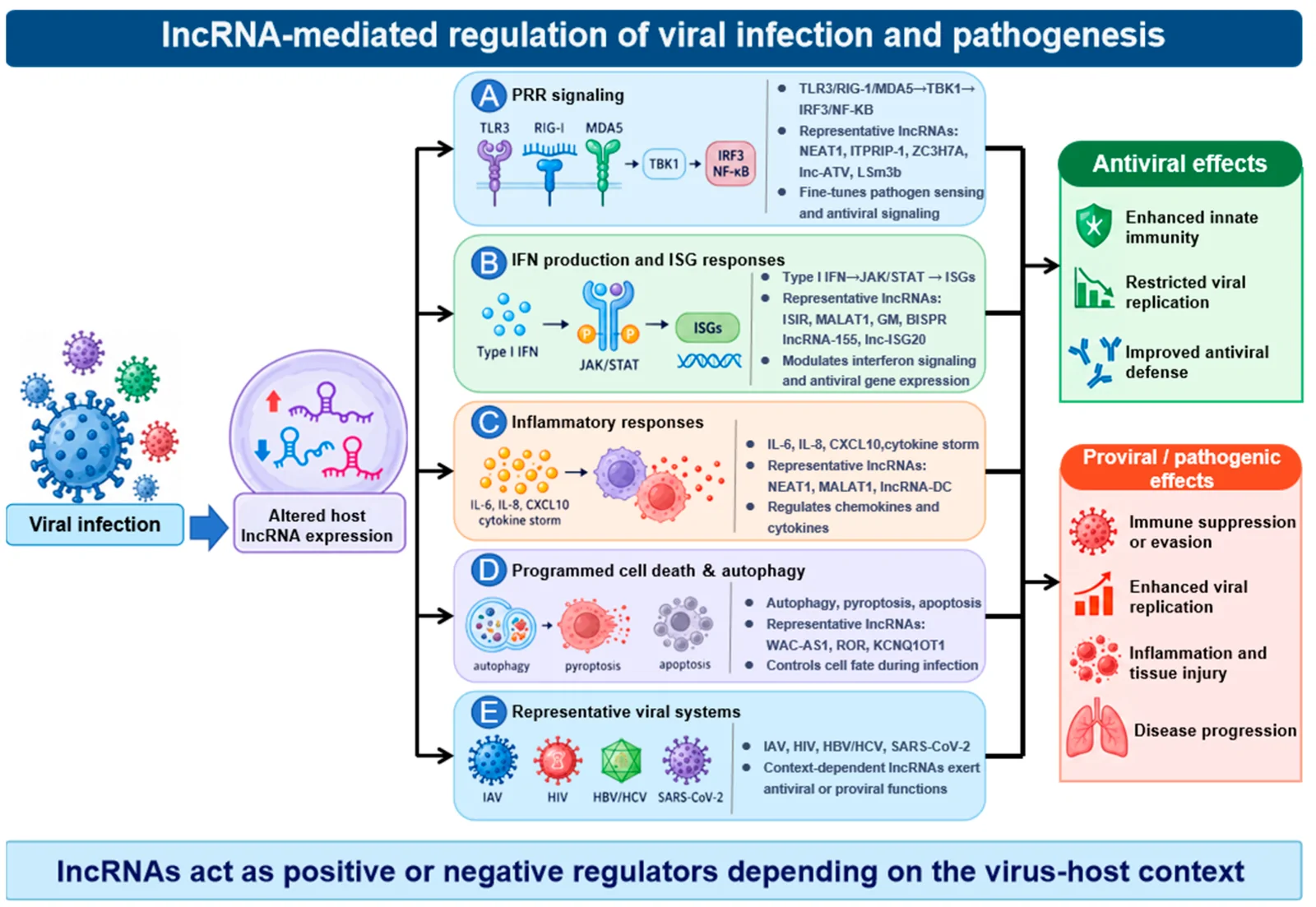
Schematic representation of the major mechanisms by which lncRNAs regulate viral infection and pathogenesis. lncRNAs modulate antiviral immunity and viral replication through PRR signaling, IFN/ISG responses, inflammatory pathways, programmed cell death, and autophagy. Depending on the virus–host context, these regulatory effects may be antiviral or pro-viral, ultimately influencing viral replication, immune responses, inflammation, and disease progression.

It is well known that lncRNAs have become an indispensable part of the host gene regulatory network, playing multifaceted roles in antiviral innate immunity. They coordinate the outcome of host–virus interactions by dynamically regulating PRR signaling, IFN components, and PCD (Figure 2). Notably, recent discoveries have shown that lncRNAs can encode functional micropeptides, expanding our understanding of the regulatory complexity in host–pathogen interactions.

Despite technological advances that have deepened our understanding of lncRNAs, many challenges remain. For instance, dissecting the function and molecular mechanisms of individual lncRNAs, as well as developing stable delivery systems suitable for therapeutic applications, are areas that require further investigation. In particular, the roles of lncRNAs in programmed cell death during viral infection—and their potential to encode biologically active micropeptides—represent priority areas for future research.

## Figures and Tables

**Table 1:** The functions of different LncRNAs.

Gene	Origin	Function	Reference
LncRNA-NEAT1	Tetrapods	enhances TLR3 stimulation-induced CXCL8 expression	[23]
LncRNA-ITPRIP-1	Homo sapiens	positively regulates the RLR signaling pathway by targeting MDA5	[24]
LncRNA-ZC3H7A	Mice	enhances RIG-I-mediated antiviral defense via TRIM25-dependent K63 ubiquitination of RIG-I	[25]
Lnc-TCONS_00113417	Duck	Participates in the regulation of IL6 and IL10	[26]
LncRNA-NEAT1	Tetrapods	LncRNA-NEAT1 regulates the IL-8 signaling pathway and cytokine expression	[27,28,29,30,31]
LncRNA-DC	Homo sapiens	modulates DC growth, apoptosis, and immune responses through the TLR9/STAT3 signaling axis	[32,33]
LncRNA-ISIR	Mice	IFN-I induces the expression of lncRNA-ISIR	[34]
LncRNA Malat1	Gnathostomes	negatively regulates type I IFN production	[35]
LncRNA-GM	Mice	binds to glutathione S-transferase M1 (GSTM1), blocking its interaction with TBK1 and reducing GSTM1-mediated S-glutathionylation of TBK1	[36]
LncRNA-ATV	Homo sapiens	modulating RIG-I function	[37]
LncRNA-LSm3b	Mice	modulating RIG-I function	[38]
LncRNA-ISG20	Homo sapiens	binding to miR-326, thereby reducing the inhibition of ISG20 mRNA translation	[39]
LncRNA-155	Homo sapiens; Mice	modulating IFN responses mediated by TLR3 and protein tyrosine phosphatase 1B	[40]
LncRNA-BISPR	Homo sapiens	through the JAK-STAT pathway, and potential diagnostic or prognostic biomarkers for HCV infection	[41,42]
lncRNA-GAS5, lncRNA-HEIH	Homo sapiens	potential diagnostic and prognostic biomarkers for HCV infection	[43]
LncRNA-EIF3J-DT	Homo sapiens	modulate tumor progression through autophagy regulation	[44]
LncRNA NBR2	Homo sapiens	modulate tumor progression through autophagy regulation	[45]
LncRNA DDIT4-AS1	Homo sapiens	modulate tumor progression through autophagy regulation	[46]
LncRNA-TUG1	Mammal	influence myocardial injury via pyroptosis or autophagic pathways	[47]
LncRNA XIST	Mammal	influence myocardial injury via pyroptosis or autophagic pathways	[48]
LncRNA Gm15834	Mice	influence myocardial injury via pyroptosis or autophagic pathways	[49]
LncRNA ROR	Homo sapiens	binds to PTBP1, destabilizes FOXP1 mRNA, and release of pyroptotic effectors	[50]
LncRNA WAC-AS1	Homo sapiens	enhancing autophagy through the miR-192-5p/Agt7 signaling axis	[51]
LncRNA KCNQ1OT1	Mammal	promotes pyroptosis via the miRNA-223-3p/NLRP3 axis, mediates autophagy induction through the miR-26a-5p/Atg12 axis, and maintaining genomic stability and regulating cellular senescence	[52,53,54]

**Table 2:** The effects of different LcnRNAs on viruses.

Gene	Origin	The Impact and Mechanism of the Virus	Reference
LncRNA-IVRPIE	Homo sapiens	promote IFN and ISGs expression to inhibit IAV replication	[67]
LINC02574	Homo sapiens	positively regulate the innate immune response to inhibit IAV replication	[68,69,70]
LncRNA-SAAL	Mice	upregulate the mRNA level of Serpina3i to inhibit IAV replication	[68,69,70]
LncRNA PCBP1-AS1	Homo sapiens	upregulate PESP protein expression to enhance IAV replication	[71]
LncRNA GAS5-P50	Homo sapiens	interacts with NOTUM and activates the Wnt/β-catenin pathway to enhance IAV replication	[72]
LncRNA-NEAT1	Tetrapods	inhibiting the expression of this inflammatory factor to promote HIV transcription	[73]
LncRNA-NKILA	Homo sapiens	regulate NF-κB to restrict HIV transcription	[74]
LncRNA-TUG1	Mammal	upregulate p21, p16, SA-β-gal activity, and proinflammatory cytokines and regulate the stability of the latent viral reservoir and HIV-associated neurocognitive disorders	[75,76,77,78]
LINC02453	Homo sapiens	bind to SEC13 to inhibits HIV-1 replication	[79]
LncRNA-NEAT1	Tetrapods	modulating the miR-9-BGH3 axis to promote HCV-induced hepatocarcinogenesis	[80]
LncRNA WAC-AS1	Homo sapiens	reinforce the autophagy induced by miR-192-5p/ATG7 to enhances HCV replication	[51]
LncRNA-HULC	Homo sapiens	stimulates HBx to activate and recruit STAT3 to the miR-539 promoter to contribute to virus-induced hepatocarcinogenesis	[81]
LncRNA-HOTAIR	Homo sapiens	Recruits transcription factor Sp1 to contribute to virus-induced hepatocarcinogenesis	[81]
LncRNA-MEG3	Homo sapiens	contribute to virus-induced hepatocarcinogenesis	[81]
LncRNA-NEAT1	Tetrapods	bind to the SARS-CoV-2 genome to participate in regulating the cytokine storm induced by COVID-19	[82]
LncRNA-MALAT1	Gnathostomes	participate in regulating the cytokine storm induced by COVID-19	[83]
LncRNA-ANRIL	Homo sapiens	potential substances for predicting severe COVID-19 patients	[84]
LncRNA-THRIL	Homo sapiens	potential substances for predicting severe COVID-19 patients	[84]
LncRNA-HOTAIRM1	Homo sapiens	may interact with the 5′ end region of the SARS-CoV-2 genome	[85]
LncRNA-PVT1	Vertebrate	may interact with the 5′ end region of the SARS-CoV-2 genome	[85]
LncRNA H19	Mammal	bind to the mRNA encoding the SARS-CoV-2 spike protein to affect the infectivity of the virus	[86]

## Data Availability

This is a review article, and no original experimental data have been generated. All information included is based on previously published studies that are appropriately referenced within the manuscript.
